# Acute and Subchronic Toxicity Study of *Euphorbia hirta* L. Methanol Extract in Rats

**DOI:** 10.1155/2013/182064

**Published:** 2013-12-10

**Authors:** Kwan Yuet Ping, Ibrahim Darah, Yeng Chen, Subramaniam Sreeramanan, Sreenivasan Sasidharan

**Affiliations:** ^1^Institute for Research in Molecular Medicine (INFORMM), Universiti Sains Malaysia, 11800 Minden, Pulau Pinang, Malaysia; ^2^School of Biological Sciences, Universiti Sains Malaysia, 11800 Minden, Penang, Malaysia; ^3^Dental Research & Training Unit, Oral Cancer Research and Coordinating Centre (OCRCC), Faculty of Dentistry, University of Malaya, 50603 Kuala Lumpur, Malaysia

## Abstract

Despite *Euphorbia hirta* L. ethnomedicinal benefits, very few studies have described the potential toxicity. The aim of the present study was to evaluate the *in vivo* toxicity of methanolic extracts of *E. hirta*. The acute and subchronic oral toxicity of *E. hirta* was evaluated in Sprague Dawley rats. The extract at a single dose of 5000 mg/kg did not produce treatment related signs of toxicity or mortality in any of the animals tested during the 14-day observation period. Therefore, the LD 50 of this plant was estimated to be more than 5000 mg/kg. In the repeated dose 90-day oral toxicity study, the administration of 50 mg/kg, 250 mg/kg, and 1000 mg/kg/day of *E. hirta* extract per body weight revealed no significant difference (*P* > 0.05) in food and water consumptions, body weight change, haematological and biochemical parameters, relative organ weights, and gross findings compared to the control group. Macropathology and histopathology examinations of all organs including the liver did not reveal morphological alteration. Analyses of these results with the information of signs, behaviour, and health monitoring could lead to the conclusion that the long-term oral administration of *E. hirta* extract for 90 days does not cause sub-chronic toxicity.

## 1. Introduction

Medicinal plants, either as an extract, pure compound or as a derivative, offer unlimited opportunities for the discovery of new drugs. Most of the natural products used in folk remedy have solid scientific evidence with regard to their biological activities. However, there is little information or evidence available concerning the possible toxicity that medicinal plants may cause to the consumers [1]. In relation to drug discovery and development, there are different weights of concern of all relevant groups such as health authorities, pharmaceutical industry, and patients which need to be taken into consideration [2]. The general public, patients and consumers are primarily interested in fast access to safe and efficient drugs, as well as in animal welfare. Based on their long-term use by humans one might expect plants used in traditional medicine to have low toxicity. Nonetheless, the latest surveys have indicated that many medicinal plants applied in traditional medicine showed adverse effects [3, 4]. Therefore, it should be emphasized that the traditional use of any plant for medicinal purposes, by no means, guarantees the safety of such plant. This raises concern about the potential toxic effects resulting from the short-term and long-term use of such medicinal plants. The data of the acute and subchronic toxicity studies on medicinal plants or preparations derived from them should be obtained in order to increase the confidence in their safety to humans, particularly for use in the development of pharmaceuticals [5]. Therefore, evaluating the toxicological effects of any medicinal plant extract intended to be used in animals or humans is a crucial part of its assessment for potential toxic effects.

*Euphorbia hirta *L., which belongs to the family Euphorbiaceae, is a small annual herb common to tropical countries. It is usually erect, slender-stemmed and spreads up to 80 cm tall, although sometimes it can be seen lying down. The plant is an annual broad-leaved herb that has a hairy stem with many branches from the base to the top. The leaves are opposite, elliptical, oblong or oblong-lanceolate, with a faintly toothed margin, and darker on the upper surface. The flowers are small, numerous, and crowded together in dense cymes (dense clusters in upper axils) about 1 cm in diameter. The stem and leaves produce a white or milky juice when cut. The plant is frequently seen occupying open waste spaces, banks of watercourses, grasslands, roadsides, and pathways [6, 7]. *E. hirta *is a very popular herb amongst practitioners of traditional medicine and is widely used as a decoction or infusion to treat various ailments including intestinal parasites, diarrhoea, peptic ulcers, heartburn, vomiting, amoebic dysentery, asthma, bronchitis, hay fever, laryngeal spasms, emphysema, coughs, colds, kidney stones, menstrual problems, sterility, and venereal diseases. Moreover, the plant is also used to treat affections of the skin and mucous membranes, including warts, scabies, tinea, thrush, aphthae, fungal afflictions, measles, and guinea-worm and as an antiseptic to treat wounds, sores, and conjunctivitis. The plant has a reputation as an analgesic to treat severe headache, toothache, rheumatism, colic, and pains during pregnancy. It is used as an antidote and pain relief of scorpion stings and snakebites. The use of the latex to facilitate the removal of thorns from the skin is common [8]. The sedative, anxiolytic, analgesic, antipyretic, and anti-inflammatory properties of *E. hirta *have been reported in the literature [9]. The leaf extract of *E. hirta *increased urine output and electrolytes in rats [10]. Furthermore, studies revealed that *E. hirta *possesses galactogenic, antianaphylactic, antimicrobial, antioxidant, anticancer, antifeedant, antiplatelet aggregation and anti-inflammatory, aflatoxin inhibition, antifertility, anthelmintic, antiplasmodial, antiamoebic, antimalarial, larvicidal, and repellent and antifeedant activities against *Plutella xylostella *[6].

Recent studies also reported the standardization of *E. hirta *methanol extract with respect to authenticity, assay, and chemical constituent analysis by Kwan et al. [11]. The results of the pharmacognostical standardization of this plant from their studies serve as a reference and help in future the identification and authentication of this plant specimen. Kwan et al. [12] also tested the genotoxicity of *E. hirta* by using *Allium cepa *assay. The results of this study confirmed that the methanol extracts of *E. hirta *exerted significant genotoxic and mitodepressive effects at 1,000 *μ*g/mL. Rajeh et al. [13] also reported preliminary oral acute toxicity of *E. hirta* leaf methanol extract. They reported that *E. hirta* leaves methanol extract exhibited mild toxic effects in mice at a dose of 5000 mg/kg body weight. Moreover, Rajeh et al. [13] also reported the cytotoxicity activity of *E. hirta *leaves, stems, flowers, and roots methanol extracts by using brine shrimp lethality assay. LC_50_ values of 1.589, 1.420, 0.206, and 0.0827 mg/mL were obtained from stems, leaves, flowers, and roots of *E. hirta*, respectively. They observed that the* E. hirta* leaves and stems might be nontoxic, and, in contrast, the flowers and roots might be toxic to the brine shrimp (*A. salina*) used in their study. Based on *E. hirta* use in traditional practices and the literature references, the present study was undertaken to evaluate the comprehensive acute and subchronic toxicity in the animal model and is reported hereunder.

## 2. Materials and Methods

### 2.1. Preparation of *Euphorbia hirta* Methanol Extract

Samples of *E. hirta *were collected from various areas in Universiti Sains Malaysia, Penang, in January 2012 and were identified by Mr. Shanmugam Vellosamy at the Herbarium School of Biological Sciences, Universiti Sains Malaysia, Pulau Pinang, Malaysia, where the voucher specimen was deposited (number USM/HERBARIUM/11215). All the samples of *E. hirta* were thoroughly rinsed with tap water and distilled water before being air-dried at room temperature for 7 days. Then, the plant sample was ground to a fine powder and soaked in absolute methanol for 4 days with frequent agitation at room temperature. The extract was filtered with Whatman paper no. 1 and the residue of fine powder was then re-soaked with a fresh portion of methanol twice for four days each time at room temperature. The filtrate was then concentrated by rotary evaporator under reduced pressure. The yield of the extract was 17.1% based on dry weight. The dried residue of plant extract was resuspended in DMSO (Sigma, USA) for further biological assay. To standardize the fingerprint of *E. hirta *extract, GC/MS chromatographic analysis was performed on a thermogas chromatograph-mass spectrometer (model Shimadzu 2010) equipped with DB-5 capillary column (30 m long. 0.25 mm i.d., film thickness 0.25 *μ*m). The column temperature program was 60°C for 0 min, with 10°C per min increases to 270°C; which was maintained for 30 min. The carrier gas was helium at a flow rate of 1 mL/min with split 10 : 1. The detector and injector temperatures were both maintained at 270°C. The quadrupole mass spectrometer was scanned over the range 28–400 amu at 1 scan s-1, with an ionising voltage of 70 eV, an ionisation current of 150* *Ma, and an ion source temperature of 200°C. In order to determine the Kovats index of the components, a mixture of alkenes (C_9_–C_24_) was added to the crude extract before injecting in the GC-MS equipment and analysed under the same conditions as above. The compounds were identified by computer searches in the commercial libraries of the National Institute of Standard and Technology (NIST) and by their Kovats retention indexes.

### 2.2. Experimental Animals

Male and female Sprague Dawley (SD) rats were used for the acute and subchronic toxicology studies. The rats were obtained from the Animal Research and Service Centre (ARASC), Universiti Sains Malaysia. The animals were acclimatized to laboratory conditions for 7 days prior to the experiments. The rats were maintained at a room temperature of 22–24°C, with a 12 h light/dark cycle. During acclimatization, the animals were housed in polycarbonate cages with a standard pellet diet and tap water *ad libitum*. The food pellets for the experimental animals were purchased from Gold Coin Holdings Sdn. Bhd. (Malaysia). All procedures in this study were performed according to the Animal Ethics Committee, Universiti Sains Malaysia, (USM/Animal Ethics Approval/2011/(74) (375)).

### 2.3. Acute Oral Toxicity Study

An acute toxicity test was performed according to the Organization of Economic Co-operation and Development (OECD) guideline 420 for testing of chemicals [14]. Rats of both sexes, aged 6–8 weeks, old were used. *E. hirta* methanol extract was dissolved in 10% Tween 20 and administered orally (only once) at a single dose of 5000 mg/kg at a rate of 20 mL/kg to both male and female rats (*n* = 12; 6 males and 6 females), whereas the control group received only 10% Tween 20 as a vehicle. After administration of *E. hirta* methanol extract, rats were observed for 24 h, with special attention given to the first 4 h and once daily further for a period of 14 days. The rats were weighed and visual observations for mortality, behavioural pattern (salivation, fur, lethargy, and sleep), changes in physical appearance, injury, pain, and signs of illness were conducted once daily during the period. At the end of the experiment, the rats were anesthetized through intraperitoneal injection of a cocktail containing ketamine (60 mg/kg) and xylazine (7.5 mg/kg). Blood samples were collected via cardiac puncture into nonheparinized and EDTA-containing tubes for biochemical and haematological analyses, respectively. After cardiac puncture, the rats were euthanized through intraperitonealinjection of a cocktail containing ketamine (80 mg/kg) and xylazine (10 mg/kg). The organs were excised, weighed, and examined macroscopically. The relative organ weight was calculated. Principal vital organs (liver, kidney, lung, heart, and spleen) were preserved in a fixation medium of 10% solution of buffered formalin for histopathological study. Haematological and biochemical analyses were performed at Gribbles Pathology (M) Sdn. Bhd., Penang, Malaysia.

### 2.4. Subchronic Oral Toxicity Study

The subchronic toxicity test was performed following the protocol described by the OECD guideline 408 for testing chemicals [15]. Rats of both sexes were randomly assigned into five groups: a control group and four treatment groups (*n* = 20; 10 males and 10 females). The *E. hirta* methanol extract was dissolved in 10% Tween 20 and administered orally on daily basis for 90 days at single doses of 50, 250, and 1000 mg/kg, while the control group received only 10% Tween 20 in distilled water. An additional group was devised as the satellite group in order to observe the reverse sign of any toxicity. The satellite group was orally administered with the extract at a daily dose of 1000 mg/kg/day for 90 days, and there was no further treatment for the following 28 days before termination of the study. The extract was freshly prepared with vehicle on daily basis. The rats were weighed and visual observations for mortality, behavioral pattern (Salivation, fur, lethargy, and sleep), changes in physical appearance, injury, pain and signs of illness were conducted once daily during that period. At the end of the experiment, all animals were anesthetized through intraperitonealinjection of a cocktail containing ketamine (60 mg/kg) and xylazine (7.5 mg/kg). Blood samples were collected via cardiac puncture into nonheparinized and EDTA-containing tubes for biochemical and haematological analyses, respectively. After cardiac puncture, the rats were euthanized by ketamine/xylazine (80 mg/kg/10 mg/kg) overdose. The organs were excised, weighed, and examined macroscopically. The relative organ weight was calculated. Principal vital organs (liver, kidney, lung, heart, and spleen) were preserved in fixation medium of 10% solution of buffered formalin for histopathological study. Haematological and biochemical analyses were performed at Gribbles Pathology (M) Sdn. Bhd., Penang, Malaysia.

### 2.5. Histopathological Study

After sacrificing the rats, parts of the liver, kidney, lung, heart, and spleen tissues were collected for histological studies. The tissues were washed in normal saline and fixed immediately in 10% formalin for a period of at least 24 h, dehydrated with alcohol, embedded in paraffin, cut into 4-5 *μ*m thick sections, and stained with haematoxylin-eosin dye for photomicroscopic observation. The microscopic features of the organs of male and female rats were compared with the control group.

### 2.6. Statistical Analysis

All values are expressed as mean ± SEM. Comparisons between groups were performed using one way analysis of variance (ANOVA) followed by Tukey's multiple comparison tests using SPSS statistical software. A *P* value of < 0.05 was considered significant.

## 3. Results

### 3.1. Preparation of *Euphorbia hirta* Methanol Extract

**Figure 1:**
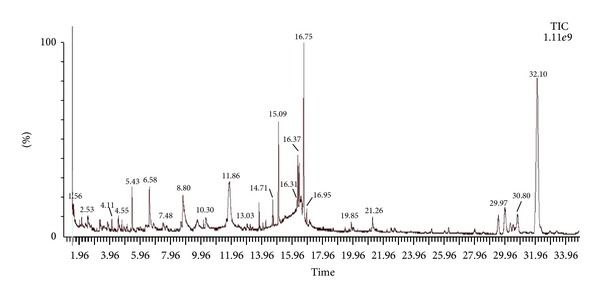
GC/MS chromatogram of *Euphorbia hirta. *

To standardize the fingerprint, ten batches of *E. hirta* samples were analysed with the developed procedure. GC/MS chromatogram of *E. hirta*, as shown in Figure 1. The peaks that existed in all the ten batches of samples were assigned as “common peaks” for the *E. hirta* extract (Figure 1). There are twenty-two “common peaks” in the fingerprint. Of these components, the 8 most intense, corresponding to 53.317% (w/w) of the *E. hirta *extract, were 2-butanone, 3,3-dimethyl-1 (methylsulfonyl)-, O-[(methylamino)carbonyl]oxime (RT (retention time): 1.599; Area: 2.225%), 2-furancarboxaldehyde, 5-(hydroxymethyl)-(RT: 6.583; area: 2.164%), 1,2,3-benzenetriol (RT: 8.795; area: 5.290%), 1,3,4,5-tetrahydroxycyclohexanecarboxylic acid (RT: 11.863; area: 9.636%), hexadecanoic acid (RT: 15.088; area: 2.916%), 9,12,15-Octadecatrienoic acid (RT: 16.754; area: 5.497%), stigmast-5-en-3-ol (RT: 29.970; 2.836%) and 9,19-cyclolanost-24-en-3-ol (RT: 32.097; area: 22.753%). The abundant presence of phytochemicals in whole plant of *E. hirta *like flavonoids, alkaloids, saponins, resins, sterols, steroids, acidic compounds, tannins, glycosides, anthraquinone, phenols and terpenoids was reported by Kader et al. [16]. Alcoholic solvents (such as methanol which was used in this study to prepare *E. hirta* extract) have been commonly used to extract polyphenols from natural sources [17]. Moreover, Pióro-Jabrucka et al. [18] also reported the presence of phenolic compounds in the *E. hirta *extract*.*

### 3.2. Acute Oral Toxicity

**Figure 2:**
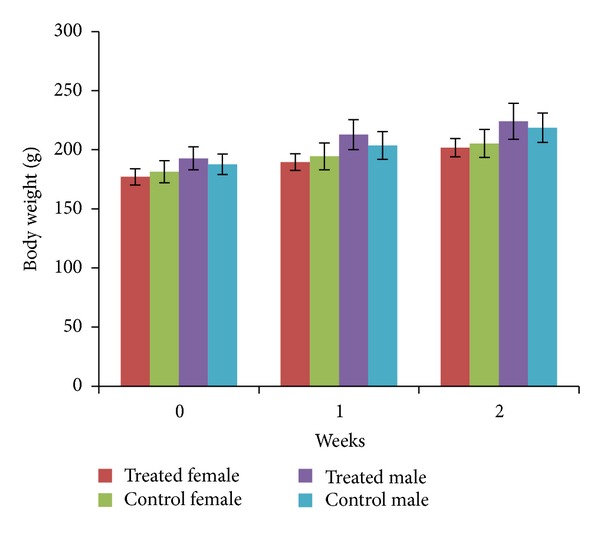
Mean body weight of rats receiving *Euphorbia hirta* extract for 14 days. Values are expressed as mean ± SEM (*n* = 6 for each group). *P values < 0.05 were considered significant using one way ANOVA followed by Tukey's multiple comparison tests. Asterisks denote significant difference compared to control.

Methanolic extract of *E. hirta* at a dose of 5000 mg/kg had no adverse effect on the behavioural responses of the tested rats up to 14 days of observation. Physical observations indicated no signs of changes in the skin, fur, eyes mucous membrane, behaviour patterns, tremors, salivation, and diarrhoea of the rats. There was no mortality observed at the tested dose nor was the weight loss in the rats affected (Figure 2). There were generally no significant differences observed in the relative organ weights in this study (Table 1). However, significant differences (*P* < 0.05) were seen in the liver and spleen in the treated groups when compared with the control group. From the present study it was seen that there was no significant change in the haematological and biochemical parameters in the *E. hirta *extract treated group compared to the normal control group (Tables 2 and 3). Gross examination at autopsy and histopathological evaluations of the various organs stained with haematoxylin and eosin revealed no significant differences (Figure 5). The LD_50_ of this plant was therefore estimated to be more than 5000 mg/kg.

### 3.3. Subchronic Oral Toxicity 

#### 3.3.1. Clinical Signs, Necropsy Findings, and Food and Water Consumption

**Figure 3:**
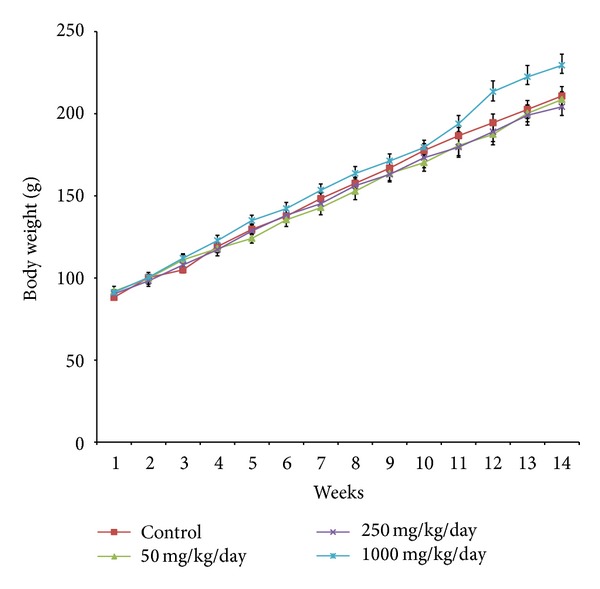
Mean body weight of male rats receiving *Euphorbia hirta* extract for 90 days. Values are expressed as mean ± SEM (*n* = 6 for each group). *P values < 0.05 were considered significant using one way ANOVA followed by Tukey's multiple comparison tests. Asterisks denote significant difference compared to control.

**Figure 4:**
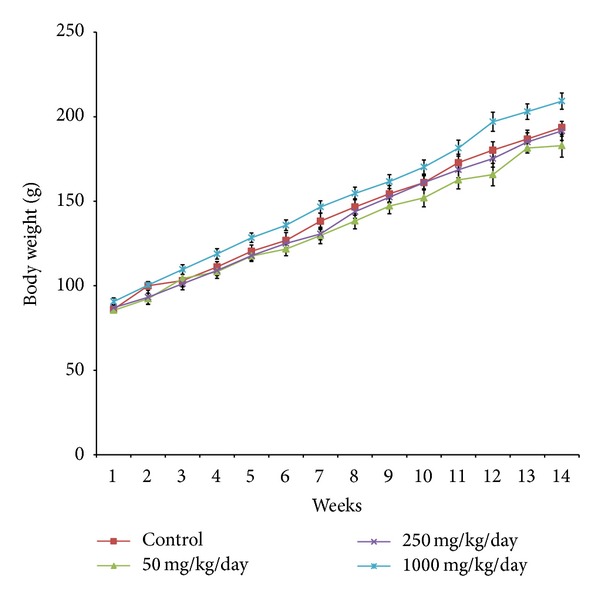
Mean body weight of female rats receiving *Euphorbia hirta* extract for 90 days. Values are expressed as mean ± SEM (*n* = 6 for each group). *P values < 0.05 were considered significant using one way ANOVA followed by Tukey's multiple comparison tests. Asterisks denote significant difference compared to control.

**Figure 5:**
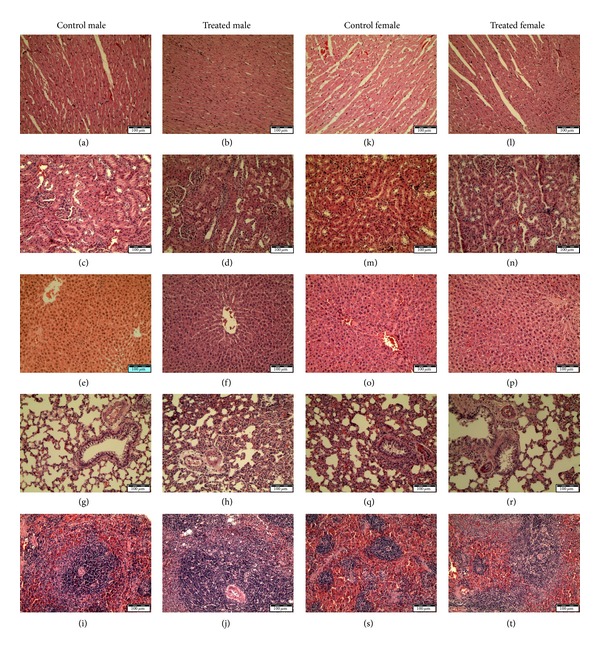
Effects of the 5000 mg/kg of *Euphorbia hirta* extract on various rat organ histomorphologies in acute oral toxicity study. (a), (b), (k), and (l): heart; (c), (d), (m), and (n): kidney; (e), (f), (o), and (p): liver; (g), (h), (q), and (r): lung; (i), (j), (s), and (t): spleen.

Daily oral administration of *E. hirta* extract for 90 days did not induce any obvious symptom of toxicity in rats of both sexes, including the highest dose tested at 1000 mg/kg body weight. No deaths or obvious clinical signs were found in any groups throughout the experimental period. Physical observation of the treated rats throughout the study indicated that none of them showed signs of toxicity in their skin, fur, eyes, mucus membrane, or behavioural changes, diarrhoea, tremors, salivation, sleep, and coma. Normal body weight gains were observed during the study period compared to the control group (Figures 3 and 4). No abnormal gross findings were observed in the necropsies of any of the rats. The food and water consumptions of the treated rats, which were measured throughout the study, were also not significantly different compared to the control rats.

#### 3.3.2. Relative Organ Weights (ROW) of Male and Female Rats

Relative organ weights of 90-day treated rats are shown in Table 4. The relative organ weight of each organ recorded at necropsy in the treatment groups did not show a significant difference (*P* > 0.05) compared to the control (Table 4).

#### 3.3.3. Haematology and Clinical Biochemistry Analysis

The effects of subchronic administration of *E. hirta *extract on haematological and biochemicalparameters are presented in Tables 5 and 6. Most haematology measures (haemoglobin, total red blood cells, red blood cells distribution width, total white blood cells, neutrophils, lymphocytes, monocytes and platelet count) in treated rats were not significantly different from the controls, with the exception of marginal variations in certain parameters (Table 5). Moreover, the *E. hirta *extract had no effect on the serum electrolytes, such as sodium, potassium, and chloride. The kidney function parameters (urea, creatinine, and uric acid) did not reveal any relevant changes following administration of *E. hirta *extract. No statistically significant differences in liver function parameters (ALT, AST, and alkaline phosphatase) were noted with the exception of marginal variations. No significant changes in total protein, globulin and albumin were noted (Table 6).

#### 3.3.4. Macropathology and Histopathology

**Figure 6:**
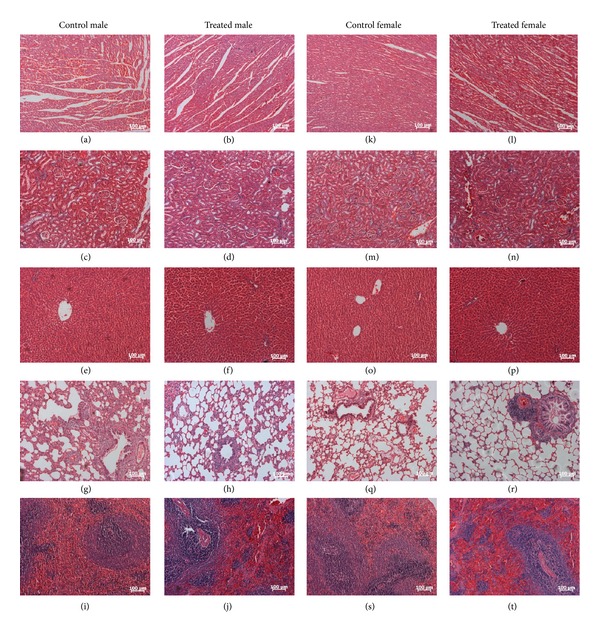
Effects of the 1000 mg/kg of *Euphorbia hirta* extract on various rat organ histomorphologies in subchronic toxicity study. (a), (b), (k), and (l): heart; (c), (d), (m), and (n): kidney; (e), (f), (o), and (p): liver; (g), (h), (q), and (r): lung; (i), (j), (s), and (t): spleen.

Macroscopic examination of the vital organs of treated animals revealed no abnormalities in the colour or texture when compared with the organs of the control group. The light microscopy examinations of the transverse section of *E. hirta *extract and organs of the control group rats are shown in Figure 6. Histopathological examination of the control group and *E. hirta* extract treated rats showed normal structure and absence of any gross pathological lesion in organs.

## 4. Discussion

For centuries, natural products, such as medicinal plants have been the basis for the treatment of various ailments [19]. In screening natural products for the pharmacological activity, assessment and evaluation of the toxic characteristics of a natural product extract, fraction, or compound are usually an initial step. Regardless of the pharmacological beneficial effects of *E. hirta*, detailed knowledge about the chronic toxicology of this famous herb is lacking. Hence, the current study was undertaken to evaluate and focus on the acute and chronic toxicity of *E. hirta *in an animal model.

During the evaluation of the toxic characteristics of medicinal plants, the determination of LD_50_ is usually an initial step to be conducted. Data from the acute toxicity study may (a) serve as the basis for classification and labelling; (b) provide initial information on the mode of toxic action of a substance; (c) help arrive at a dose of a new compound; (d) help in dose determination in animal studies; and (e) help determine LD_50_ values that provide many indices of potential types of drug activity [5]. Moreover, if a high dose (e.g., 5,000 mg/kg) is found to be survivable, no further acute testing will be conducted [20]. In this study, *E. hirta* extract at a dose of 5000 mg/kg had no adverse effect on the tested rats up to 14 days of observation. The significant changes in the relative weights of the liver and spleen in the presence of *E. hirta* extract may indicate that the extract might have toxic potential on the liver and spleen. However, it could be argued that these changes may not be toxicologically significant, as they were not corroborated by the biochemical and histomorphology findings. Therefore, this study indicates that *E. hirta* extract does not cause acute toxicity effects at the dose tested and with an LD_50_ value greater than 5000 mg/kg. In principle, the limit test method is not intended for determining a precise LD_50_ value, but it serves as a suggestion for classifying the crude extract based on the expectation at which dose level the animals are expected to survive [21]. According to the chemical labelling and classification of acute systemic toxicity recommended by OECD, the crude extract of *E. hirta* extract was assigned class 5 status (LD_50_ > 5000 mg/kg), which was the lowest toxicity class. According to the study by Kennedy et al. [22] substances with LD_50_ values higher than 5000 mg/kg by oral route are regarded as being safe or practically nontoxic. However, many medicinal plants have also been reported to be toxic to both humans and animals. Therefore, it should be emphasized that the traditional use of any plant for medicinal purposes, by no means, guarantees the safety of such plant. Furthermore, the data of the acute and subchronic toxicity studies on medicinal plants or preparations derived from them should be obtained in order to increase the confidence in its safety to humans, particularly for use in the development of pharmaceuticals [5]. Choosing the appropriate tests and dosing regimens that will demonstrate an adequate margin of exposure is a critical step in establishing human safety. Since no toxic effects were found during the acute toxicity study, further evaluation was conducted to evaluate the subchronic toxicity of *E. hirta* extract up to 90 days in rats to prepare the comprehensive toxicology data of this ancient medicinal plant.

Subchronic studies assess the undesirable effects of continuous or repeated exposure of plant extracts or compounds over a portion of the average life span of experimental animals, such as rodents. Specifically, they provide information on target organ toxicity and are designed to identify noobservable adverse effect level [20]. Subchronic evaluation can also help to determine appropriate dose regimens for longer-term studies. Consequently, in this study the subchronic toxicity *E. hirta* extract was evaluated in rats at doses of 50–1000 mg/kg/day for 90 days. Consideration should be given to an additional satellite group treated with top dose group for observation of reversibility, persistence or delayed occurrence of systemic toxic effects and recovery from toxic effects, for at least 14 days after treatment. Satellite groups are groups of animals included in the design and conduct of a toxicity study. This group will be treated and housed under the conditions identical to those of the animals from main experimental group but used mainly for toxicokinetics [15]. The satellite group in this study was orally administered with the *E. hirta* extract at a daily dose of 1000 mg/kg/day for 90 days, and no further treatment for the following 28 days before the termination of the study. However, in the satellite group no reversibility or persistence of any toxic effects was observed in this study which further verified nontoxic nature of *E. hirta* extract tested in this study.

Administration of extracts of *E. hirta *for 90 days produced no clinical signs for toxicity or mortality in either sex. In addition, the treated rats did not show any significant alteration in water or food consumption (data not shown). Significant reduction in food and water intake is suggested as being responsible for the observed decrement in body weight gain. Loss of appetite is often synonymous with weight loss due to disturbances in carbohydrate, protein or fat metabolisms [23]. Moreover, at higher doses, crude plant extracts may metabolise to a toxic end product, which could interfere with gastric function and decreased food conversion efficiency [24]. Interestingly, the food and water intakes were found to be unaltered during the 90-day treatment period when compared to a control group in this study. Furthermore, the diets and water were well-accepted by the rats treated with *E. hirta* extract suggesting the extract did not possibly cause any alterations in carbohydrate, protein or fat metabolism in these experimental animals. It also shows that the *E. hirta* extract did not adversely interfere with the nutritional benefits, such as weight gain and stability of the appetite, which are expected for animals that are continually supplied with food and water *ad libitum*. Also, the extract is safe by oral route in relation to its folkloric practice in the administration of medicinal herbs through oral route to treat the patients. Therefore, *E. hirta* extract can be considered as non-toxic up to the said dose.

The body weight changes serve as a sensitive indication of the general health status of animals [25]. However, weight gains were observed in all animals administered with *E. hirta* extract. It can be stated that the *E. hirta* extract did not interfere with the normal metabolism of animals as corroborated by the nonsignificant difference from animals in the vehicle control group. The significant increment in food and water intake is considered as being responsible for the increment in body weight gain. As mentioned earlier the loss of appetite is often synonymous with weight loss due to disturbances in the metabolism of carbohydrate, protein, or fat [26]. Therefore, the normal food and water intake (*P* > 0.05) without loss of appetite are suggested as being responsible for the observed increment in body weight in this study. In addition, the observed increase in body weight could be attributed to the nutritive components in the *E. hirta* extract [26, 27]. Interestingly, it was noted that the dose 1000.00 mg/kg/day further increases the body weight in both male and female rats after the 12th week of treatment with *E. hirta *extract. Fatness is characterized by increased adipose tissue mass that results from both increased fat cell number and increased fat cell size. Adipose tissue is a dynamic organ that plays an important role in energy balance and changes in mass according to the metabolic requirements of the organism [28]. Moreover, it was reported that excess energy intake and reduced energy expenditure result in abnormal excessive growth of white adipose tissue (WAT), which can lead to the development of obesity [17, 29]. The current research findings have suggested that *E. hirta *extract with the dose 1000.00 mg/kg/day may not prevent the accumulation of WAT in rats after the 12th week of treatment. However, further mechanism studies are needed to clarify the obesity effects of *E. hirta *extract after the 12th week of treatment.

Similarly, no significant changes in the weights of the heart, liver, spleen, kidneys, or lungs were observed, suggesting that administration of *E. hirta* extract at the subchronic oral doses had no effect on the normal growth. The usefulness of weighing organs in toxicity studies includes their sensitivity to predict toxicity, enzyme induction, physiologic perturbations, and acute injury; it is frequently a target organ of toxicity; it correlates well with histopathological changes; there is little interanimal variability; historical control range data are available [30]. The relative organs weights have been observed in toxicity studies to be a relatively sensitive indicator for particular organs, and, thereafter, define toxicity as significant changes observed in those particular organs [31]. The results of this study revealed that the essential organs, such as heart, liver, spleen, kidneys, and lungs, neither were adversely affected nor showed clinical signs for toxicity throughout the treatment. Since there was no reduction in body and relative organ weights of the treated animals at any of the doses tested, we concluded that the extract is nontoxic to the analysed organs.

The serum haematology and clinical biochemistry analyses were done to evaluate the possible alterations in hepatic and renal functions influenced by the extracts. Liver and kidney function analysis is very important in the toxicity evaluation of drugs and plant extracts as they are both necessary for the survival of an organism [32]. High levels of ALT, AST, and alkaline phosphatase are reported in liver diseases or hepatotoxicity [33]. The nonsignificant changes in ALT, AST, and alkaline phosphatase in both male and female rats at all doses suggest that subchronic administration of *E. hirta* extract does not affect the hepatocyte function in the rats. A decrease in total protein, albumin, and globulin is a sign of the reduced synthetic function of the liver or might be due to impaired hepatocellular function. Low serum albumin content may suggest infection or continuous loss of albumin [34, 35]. Thus, the insignificant change in serum concentration of total protein, albumin and globulin in the *E. hirta* extract treated and control group further confirms that the extract does not damage the hepatocellular or secretory functions of the liver at any of the doses tested. Renal dysfunction can be assessed by concurrent measurements of urea, creatinine and uric acid and their normal levels reflect at reduced likelihood of renal problems [36]. In the present study, changes in plasma urea, creatinine, and uric acid levels in *E. hirta* extract treated groups showed non-significant differences indicating a normal renal function.

Evaluation of haematological parameters can be used to determine the extent of the deleterious effect of *E. hirta* extract on the blood of an animal. It can also be used to explain blood relating functions of a plant extract or its products [37]. Furthermore, such analysis is relevant to risk evaluation as changes in the haematological system have higher predictive value for human toxicity when the data are translated from animal studies [38]. A haemogram was undertaken for all the *E. hirta* extract treated and control groups and the results show no significant effects. The non-significant effect of the extract on total red blood cells, mean corpuscular volume, mean corpuscular Hb, and platelets indicates that the *E. hirta *extract does not affect the erythropoiesis, morphology, or osmotic fragility of the red blood cells [39]. Leukocytes are the first line of cellular defence that respond to infectious agents, tissue injury, or inflammatory process. Furthermore, no significant changes were observed in the neutrophils, lymphocytes, and monocytes in the *E. hirta*, which further confirmed the above findings. A normal haematological profile of *E. hirta* extract treated groups also further justified the non-toxic nature of *E. hirta* extract.

The macroscopic examinations of the organs of rats treated with various doses of *E. hirta* extract did not show any changes in colour compared with control group rats' organs. Hypertrophy of organs is first hand indication of toxicity of chemical or biological substance. However, no hypertrophy of organs was observed in this study amongst all the groups studied. In addition, the microscopic examination revealed that none of the organs from the extract treated rats showed any alteration in cell structure or any unfavourable effects when viewed under the light microscope using multiple magnification powers. No pathologies were recorded in the histological sections of the vital organs (heart, liver, spleen, kidney, and lung) of the control group. Generally, any damage to the parenchymal liver cells results in elevations of both transaminases in the blood [40]. Thus, the non-significant increases observed in ALT, AST, and alkaline phosphatase activities strongly suggest that the subchronic administration of *E. hirta* extract did not alter the hepatocytes and, consequently, the metabolism of the rats as observed in the histopathology observations of liver tissue. Equally, there was also no significant increase in urea, creatinine, and uric acid in the subchronic administration of *E. hirta* extract when compared to the control group. Any rise in urea, creatinine and uric acid levels is only observed if there is marked damage to functional nephrons [41]. This finding was further confirmed by histopathological observations of the kidney tissue in this study. Therefore, the results recorded in this study demonstrate that the *E. hirta* extract did not alter the liver or renal function and further support the non-toxic nature of *E. hirta* extract.

## 5. Conclusion 

In light of these findings, we may conclude that *E. hirta* extract is not toxic in all the doses studied herein and did not produce any toxic signs or evident symptoms at acute and subchronic oral toxicity. *E. hirta* extract did not cause any lethality or produce any remarkable histopathological signs or serum chemical alteration. The preliminary results suggest promising alternatives for exploring therapeutic and pharmaceutical interest in *E. hirta* extract with a reduction of possible adverse effects. Further studies to determine the effects of *E. hirta* extract on an animal foetus, on pregnant animals, and their reproductive capacity are needed to complete the safety profile of this herb.

## Figures and Tables

**Table 1:** Relative organ weights of rats receiving single dose of *Euphorbia hirta* extract for 14 days.

Organs	Treatment	
	Control	*Euphorbia hirta* extracts 5000 mg/kg
Male		
Heart	0.45 ± 0.02	0.42 ± 0.02
Liver	3.52 ± 0.13	3.15 ± 0.10*
Spleen	0.30 ± 0.02	0.36 ± 0.01*
Kidneys	0.83 ± 0.02	0.85 ± 0.02
Lungs	0.67 ± 0.03	0.63 ± 0.06
Female		
Heart	0.46 ± 0.02	0.44 ± 0.02
Liver	3.69 ± 0.16	3.56 ± 0.15
Spleen	0.42 ± 0.04	0.38 ± 0.02
Kidneys	0.90 ± 0.07	0.81 ± 0.04
Lungs	0.82 ± 0.03	0.83 ± 0.05

Values are expressed as mean ± SEM (*n* = 6 for each group).

Relative organ weight was calculated as (organ weight/body weight) × 100%

*P values < 0.05 were considered significant using one-way ANOVA followed by Tukey's multiple comparison tests. Asterisks denote significant difference compared to control.

**Table 2:** Effect of *Euphorbia hirta* extract on haematological parameters in acute oral toxicity study.

	Unit	Treatment	
		Control	*Euphorbia hirta* extracts 5000 mg/kg
Male			
Haemoglobin	g/L	154.33 ± 1.96	150.17 ± 2.57
Total red blood cells	10^12^/L	7.83 ± 0.15	8.14 ± 0.27
Packed cells volume	L/L	0.46 ± 0.00	0.46 ± 0.00
Mean corpuscular volume	fL	59.00 ± 0.77	56.17 ± 1.19
Mean corpuscular Hb	pg	19.67 ± 0.21	18.67 ± 0.56
Mean corpuscular Hb conc	g/L	334.50 ± 5.89	330.17 ± 8.67
Red blood cells distribution width	%	14.37 ± 0.79	14.57 ± 1.044
Total white blood cells	10^9^/L	7.27 ± 0.97	9.02 ± 1.70
Neutrophils	%	20.83 ± 3.03	27.33 ± 3.27
Lymphocytes	%	73.33 ± 2.28	69.33 ± 3.17
Monocytes	%	3.00 ± 0.73	2.00 ± 0.37
Platelet count	10^9^/L	866.00 ± 120.33	1140.33 ± 50.64
Female			
Haemoglobin	g/L	141.17 ± 5.52	142.83 ± 4.90
Total red blood cells	10^12^/L	7.48 ± 0.26	7.28 ± 0.18
Packed cells volume	L/L	0.41 ± 0.03	0.44 ± 0.011
Mean corpuscular volume	fL	55.33 ± 0.01	60.50 ± 1.18*
Mean corpuscular Hb	pg	19.00 ± 0.26	19.67 ± 0.42
Mean corpuscular Hb conc	g/L	341.83 ± 6.67	324.17 ± 6.89
Red blood cells distribution width	%	12.30 ± 0.22	13.33 ± 1.11
Total white blood cells	10^9^/L	8.93 ± 0.43	9.03 ± 0.97
Neutrophils	%	19.17 ± 3.05	16.00 ± 1.84
Lymphocytes	%	74.33 ± 3.34	79.83 ± 1.30
Monocytes	%	4.50 ± 0.99	3.67 ± 1.085
Platelet count	10^9^/L	844.67 ± 147.75	995.67 ± 55.01

Values are expressed as mean ± SEM (*n* = 6 for each group).

*P values < 0.05 were considered significant using one way ANOVA followed by Tukey's multiple comparison tests. Asterisks denote significant difference compared to control.

**Table 3:** Effect of *Euphorbia hirta* extract on biochemical parameters in acute oral toxicity study.

	Unit	Treatment	
		Control	*Euphorbia hirta *extracts 5000 mg/kg
Male			
Sodium	mmol/L	140.83 ± 0.70	140.83 ± 0.60
Potassium	mmol/L	4.37 ± 0.29	4.75 ± 0.35
Chloride	mmol/L	102.83 ± 0.83	104.50 ± 1.06
Urea	mmol/L	5.65 ± 0.52	6.083 ± 0.36
Creatinine	*μ*mol/L	24.17 ± 2.30	25.67 ± 3.21
Uric acid	mmol/L	0.15 ± 0.03	0.17 ± 0.03
Total protein	g/L	61.50 ± 1.82	62.67 ± 0.95
Albumin	g/L	29.67 ± 0.84	31.67 ± 0.67
Globulin	g/L	31.83 ± 1.17	31.00 ± 1.18
Albumin/globulin ratio		0.93 ± 0.03	1.02 ± 0.05
Alkaline phosphatase	U/L	201.83 ± 41.45	231.00 ± 11.90
AST	U/L	196.00 ± 7.31	184.33 ± 19.22
ALT	U/L	44.67 ± 1.45	49.167 ± 3.65
Female			
Sodium	mmol/L	140.00 ± 0.82	139.83 ± 0.91
Potassium	mmol/L	4.27 ± 0.19	4.33 ± 0.28
Chloride	mmol/L	105.17 ± 1.35	102.67 ± 1.23
Urea	mmol/L	8.12 ± 0.59	7.38 ± 0.61
Creatinine	*μ*mol/L	36.33 ± 6.03	33.33 ± 1.31
Uric acid	mmol/L	0.11 ± 0.00	0.15 ± 0.03
Total protein	g/L	62.33 ± 1.43	64.50 ± 1.80
Albumin	g/L	31.50 ± 0.67	32.33 ± 1.65
Globulin	g/L	30.83 ± 1.51	32.17 ± 1.30
Albumin/globulin ratio		1.02 ± 0.05	1.00 ± 0.07
Alkaline phosphatase	U/L	142.17 ± 24.23	194.83 ± 15.74
AST	U/L	139.83 ± 17.18	202.17 ± 22.27
ALT	U/L	32.67 ± 2.96	46.33 ± 1.86

Values are expressed as mean ± SEM (*n* = 6 for each group).

*P values < 0.05 were considered as significant using one way ANOVA followed by Tukey's multiple comparison tests. Asterisks denote significant difference compared to control.

**Table 4:** Relative organ weights of rats treated with *Euphorbia hirta* extract in subchronic toxicity study.

Organs	Control	*Euphorbia hirta *			
		50 mg/kg	250 mg/kg	1000 mg/kg^a^	1000 mg/kg^b^
Male					
Heart	0.44 ± 0.01	0.43 ± 0.02	0.39 ± 0.01	0.41 ± 0.01	0.42 ± 0.01
Liver	2.96 ± 0.08	3.04 ± 0.15	3.02 ± 0.05	2.98 ± 0.07	3.07 ± 0.09
Spleen	0.29 ± 0.01	0.28 ± 0.02	0.27 ± 0.01	0.26 ± 0.02	0.27 ± 0.01
Kidneys	0.72 ± 0.01	0.75 ± 0.01	0.71 ± 0.02	0.73 ± 0.02	0.67 ± 0.03
Lungs	0.90 ± 0.08	0.81 ± 0.07	0.83 ± 0.1	0.72 ± 0.03	0.73 ± 0.04
Female					
Heart	0.46 ± 0.02	0.45 ± 0.02	0.45 ± 0.02	0.45 ± 0.01	0.42 ± 0.01
Liver	3.11 ± 0.14	3.35 ± 0.07	3.26 ± 0.02	3.32 ± 0.10	3.27 ± 0.07
Spleen	0.36 ± 0.02	0.32 ± 0.01	0.30 ± 0.02	0.30 ± 0.02	0.30 ± 0.01
Kidneys	0.75 ± 0.02	0.78 ± 0.02	0.75 ± 0.01	0.75 ± 0.04	0.70 ± 0.04
Lungs	0.79 ± 0.04	0.93 ± 0.11	0.88 ± 0.03	0.87 ± 0.05	0.70 ± 0.01

Values are expressed as mean ± SEM (*n* = 10 for each group).

Relative organ weight was calculated as (organ weight/body weight) × 100%.

^a^A group was treated with methanol extract of *Euphorbia hirta *at 1000 mg/kg/day for 90 days.

^b^A satellite group was treated with the methanol extract of *Euphorbia hirta *at 1000 mg/kg/day for 90 days followed by no treatment for 28 days.

*P values < 0.05 were considered significant using one way ANOVA followed by Tukey's multiple comparison tests. Asterisks denote significant difference compared to control.

**Table 5:** Effect of *Euphorbia hirta* extract on hematological parameters in subchronic toxicity study.

	Unit	Control	*Euphorbia hirta *			
			50 mg/kg	250 mg/kg	1000 mg/kg^a ^	1000 mg/kg^b^
Male						
Haemoglobin	g/L	144.30 ± 2.91	143.00 ± 3.36	151.40 ± 4.12	148.80 ± 3.35	138.30 ± 4.92
Total red blood cells	10^12^/L	7.84 ± 0.18	7.75 ± 0.18	8.22 ± 0.26	8.14 ± 0.18	7.55 ± 0.27
Packed cell volume	L/L	0.45 ± 0.01	0.44 ± 0.01	0.46 ± 0.01	0.47 ± 0.01	0.44 ± 0.01
Mean corpuscular volume	fL	57.70 ± 0.45	57.00 ± 0.65	56.50 ± 0.70	57.30 ± 0.73	58.60 ± 0.99
Mean corpuscular Hb	pg	18.50 ± 0.17	18.50 ± 0.22	18.40 ± 0.16	18.30 ± 0.26	18.30 ± 0.34
Mean corpuscular Hb conc	g/L	319.60 ± 2.29	323.80 ± 1.81	327.00 ± 2.05	319.50 ± 3.28	313.30 ± 3.08
Red blood cells Distribution width	%	15.55 ± 0.39	15.32 ± 0.32	15.47 ± 0.45	16.96 ± 0.34	16.24 ± 0.40
Total white blood cells	10^9^/L	5.00 ± 0.76	4.99 ± 0.87	5.97 ± 0.91	4.77 ± 0.26	5.72 ± 0.75
Neutrophils	%	26.90 ± 2.05	32.70 ± 1.67	28.60 ± 1.11	31.7 ± 1.91	28.00 ± 2.67
Lymphocytes	%	69.20 ± 2.22	62.00 ± 1.78	67.30 ± 1.41	64.8 ± 2.25	66.50 ± 2.63
Monocytes	%	2.70 ± 0.40	4.10 ± 0.77	3.60 ± 0.65	2.10 ± 0.41	3.80 ± 0.36
Platelet count	10^9^/L	926.60 ± 42.34	980.80 ± 51.44	1005.20 ± 37.24	968.70 ± 27.91	947.20 ± 48.18
Female						
Haemoglobin	g/L	143.70 ± 4.05	140.50 ± 2.95	142.30 ± 3.45	144.80 ± 4.87	142.70 ± 4.24
Total red blood cells	10^12^/L	7.60 ± 0.23	7.48 ± 0.16	7.45 ± 0.17	7.65 ± 0.26	7.31 ± 0.23
Packed cell volume	L/L	0.44 ± 0.01	0.44 ± 0.01	0.44 ± 0.01	0.46 ± 0.01	0.45 ± 0.01
Mean corpuscular volume	fL	58.30 ± 0.63	58.20 ± 0.79	59.10 ± 0.60	60.20 ± 0.70	62.20 ± 1.07
Mean corpuscular Hb	pg	18.80 ± 0.20	18.70 ± 0.15	19.20 ± 0.20	18.90 ± 0.10	19.60 ± 0.16
Mean corpuscular Hb conc	g/L	324.40 ± 1.67	323.30 ± 3.96	323.30 ± 1.71	314.90 ± 3.02	314.90 ± 4.23
Red blood cell distribution width	%	14.01 ± 0.30	13.48 ± 0.19	13.19 ± 0.22	14.8 ± 0.25	15.07 ± 0.38
Total white blood cells	10^9^/L	6.53 ± 1.54	7.30 ± 1.19	5.19 ± 0.73	6.33 ± 1.01	8.09 ± 1.03
Neutrophils	%	24.50 ± 1.86	22.20 ± 3.34	23.00 ± 1.80	28.50 ± 4.43	20.30 ± 1.94
Lymphocytes	%	69.90 ± 1.64	71.60 ± 3.28	71.00 ± 2.13	65.50 ± 4.80	74.30 ± 2.26
Monocytes	%	4.40 ± 0.43	5.80 ± 0.80	5.00 ± 0.60	3.70 ± 0.58	3.90 ± 0.55
Platelet count	10^9^/L	890.40 ± 68.48	914.20 ± 24.01	931.30 ± 50.95	831.50 ± 86.06	927.10 ± 33.27

Values are expressed as mean ± SEM (*n* = 10 for each group).

^a^A group was treated with methanol extract of *Euphorbia hirta *at 1000 mg/kg/day for 90 days.

^b^A satellite group was treated with the methanol extract of *Euphorbia hirta *at 1000 mg/kg/day for 90 days followed by no treatment for 28 days.

*P values < 0.05 were considered significant using one way ANOVA followed by Tukey's multiple comparison tests. Asterisks denote significant difference compared to control.

**Table 6:** Effect of *Euphorbia hirta* extract on biochemical parameters in subchronic toxicity study.

	Unit	Control	*Euphorbia hirta *			
			50 mg/kg	250 mg/kg	1000 mg/kg^a^	1000 mg/kg^b^
Male						
Sodium	mmol/L	143.20 ± 0.49	142.80 ± 0.85	141.90 ± 0.59	142.20 ± 0.51	142.60 ± 0.43
Potassium	mmol/L	4.13 ± 0.09	4.42 ± 0.14	4.03 ± 0.123	4.29 ± 0.19	4.63 ± 0.16
Chloride	mmol/L	103.20 ± 0.44	102.70 ± 0.62	102.90 ± 0.66	102.00 ± 0.71	103.10 ± 0.95
Urea	mmol/L	6.98 ± 0.26	7.41 ± 0.42	7.12 ± 0.31	7.06 ± 0.29	7.12 ± 0.35
Creatinine	*μ*mol/L	30.30 ± 0.72	25.50 ± 1.93	26.30 ± 1.61	29.70 ± 1.08	30.70 ± 1.19
Uric acid	mmol/L	0.10 ± 0.00	0.11 ± 0.01	0.14 ± 0.04	0.12 ± 0.02	0.11 ± 0.01
Total protein	g/L	65.70 ± 1.10	67.00 ± 1.33	65.60 ± 1.21	67.00 ± 1.46	65.40 ± 1.16
Albumin	g/L	30.50 ± 0.67	28.40 ± 0.88	29.30 ± 0.94	30.60 ± 0.72	30.70 ± 0.56
Globulin	g/L	35.20 ± 0.90	38.60 ± 1.76	36.30 ± 0.86	36.40 ± 0.90	34.70 ± 1.14
Albumin/globulin ratio		0.88 ± 0.03	0.76 ± 0.05	0.80 ± 0.03	0.85 ± 0.02	0.90 ± 0.04
Alkaline phosphatase	U/L	208.10 ± 7.17	256.70 ± 13.41	269.30 ± 17.12	230.60 ± 14.23	237.50 ± 21.25
AST	U/L	232.10 ± 7.95	270.40 ± 23.32	211.90 ± 8.40	222.30 ± 7.63	199.40 ± 15.35
ALT	U/L	56.20 ± 2.30	55.10 ± 1.49	57.70 ± 2.76	65.80 ± 4.77	63.40 ± 4.92
Female						
Sodium	mmol/L	141.20 ± 0.66	139.90 ± 0.74	138.90 ± 0.53	140.00 ± 0.61	139.90 ± 0.43
Potassium	mmol/L	4.07 ± 0.12	4.28 ± 0.00	4.13 ± 0.18	4.09 ± 0.09	4.37 ± 0.18
Chloride	mmol/L	103.30 ± 0.67	102.80 ± 1.03	102.30 ± 0.56	103.30 ± 1.11	103.30 ± 0.73
Urea	mmol/L	6.93 ± 0.29	5.83 ± 0.15	6.25 ± 0.35	6.88 ± 0.38	7.38 ± 0.36
Creatinine	*μ*mol/L	29.70 ± 1.61	27.00 ± 0.80	28.10 ± 1.16	36.10 ± 1.59	36.60 ± 2.35
Uric acid	mmol/L	0.12 ± 0.00	0.12 ± 0.01	0.14 ± 0.02	0.13 ± 0.01	0.13 ± 0.01
Total protein	g/L	68.00 ± 1.42	69.20 ± 1.61	71.50 ± 2.17	69.40 ± 1.65	67.40 ± 0.90
Albumin	g/L	30.20 ± 1.08	30.40 ± 0.60	30.80 ± 0.85	31.70 ± 1.25	33.40 ± 0.64
Globulin	g/L	37.80 ± 1.31	38.80 ± 1.77	40.70 ± 1.97	37.70 ± 1.82	34.00 ± 1.02
Albumin/globulin ratio		0.81 ± 0.05	0.79 ± 0.04	0.76 ± 0.04	0.86 ± 0.06	0.97 ± 0.05
Alkaline phosphatase	U/L	178.00 ± 11.08	221.60 ± 16.48	214.00 ± 23.56	142.50 ± 9.28	153.80 ± 25.83
AST	U/L	236.6 ± 12.50	216.5 ± 13.91	225.5 ± 10.62	210.7 ± 8.56	187 ± 7.60
ALT	U/L	53.1 ± 2.9	44.7 ± 2.21	42.4 ± 3.4	47.4 ± 3.27	47.7 ± 1.80

Values are expressed as mean ± SEM (*n* = 10 for each group).

^a^A group was treated with methanol extract of *Euphorbia hirta *at 1000 mg/kg/day for 90 days.

^b^A satellite group was treated with the methanol extract of *Euphorbia hirta *at 1000 mg/kg/day for 90 days followed by no treatment for 28 days.

*P values < 0.05 were considered significant using one way ANOVA followed by Tukey's multiple comparison tests. Asterisks denote significant difference compared to control.
